# The prognostic value of radiological and pathological lymph node status in patients with cervical cancer who underwent neoadjuvant chemotherapy and followed hysterectomy

**DOI:** 10.1038/s41598-023-49539-7

**Published:** 2024-01-24

**Authors:** Jianghua Lou, Xiaoxian Zhang, Jinjin Liu, Linxiao Dong, Qingxia Wu, LiangLiang Yan, Chunmiao Xu, Qingxia Wu, Meiyun Wang

**Affiliations:** 1 Department of Medical Imaging, Henan Provincial People’s Hospital, People’s Hospital of Zhengzhou University, People’s Hospital of Henan University, No. 7 Weiwu Road, Zhengzhou, 450003 Henan China; 2 Department of Radiology, The Affiliated Cancer Hospital of Zhengzhou University, Henan Cancer Hospital, Zhengzhou, Henan China; 3 Beijing United Imaging Research Institute of Intelligent Imaging, United Imaging Intelligence (Beijing) Co., Ltd., Beijing, China; 4 Laboratory of Brain Science and Brain-Like Intelligence Technology, Institute for Integrated Medical Science and Engineering, Henan Academy of Sciences, Zhengzhou, Henan China

**Keywords:** Cervical cancer, Cancer imaging

## Abstract

To investigate the prognostic value of lymph node status in patients with cervical cancer (CC) patients who underwent neoadjuvant chemotherapy (NACT) and followed hysterectomy. Patients in two referral centers were retrospectively analyzed. The baseline tumor size and radiological lymph node status (LNr) were evaluated on pre-NACT MRI. Tumor histology, differentiation and pathological lymph node status (LNp) were obtained from post-operative specimen. The log-rank test was used to compare survival between patient groups. Cox proportional hazards regression models were employed to estimate the hazard ratio (HR) of various factors with progression-free survival (PFS) and overall survival (OS). A total of 266 patients were included. Patients with 2018 FIGO IIIC showed worse PFS compared to those with FIGO IB-IIB (p < 0.001). The response rate in patients with LNp(−) was 64.1% (134/209), significantly higher than that of 45.6% (26/57) in patients with LNp( +) (p = 0.011). Multivariate Cox analysis identified the main independent predictors of PFS as LNp( +) (HR = 3.777; 95% CI 1.715–8.319), non-SCC (HR = 2.956; 95% CI 1.297–6.736), poor differentiation (HR = 2.370; 95% CI 1.130–4.970) and adjuvant radiation (HR = 3.266; 95% CI 1.183–9.019). The interaction between LNr and LNp regarding PFS were significant both for univariate and multivariate (P = 0.000171 and 1.5357e^−7^ respectively). In patients with LNr( +), a significant difference in PFS was observed between patients with LNp(−) and LNp( +) (p = 0.0027). CC patients with FIGO 2018 stage IIIC who underwent NACT and followed hysterectomy had worse PFS compared to those with IB-IIB. LNp( +), non-SCC, poor differentiation and adjuvant radiation were independent risk factors for PFS. The adverse prognostic value of LNp( +) was more significant in patients with LNr( +).

## Introduction

Cervical cancer (CC) is the fourth leading cause of cancer-related deaths among women globally^1^. The management of CC is influenced by local traditions^2^. The standard treatment for locally advanced cervical cancer (LACC) is concurrent chemotherapy and radiotherapy (CCRT) plus brachytherapy^3^. Controversies exist regarding the optimal management of LACC, with recent focus on the application of neoadjuvant chemotherapy (NACT) before surgery or CCRT^4^. NACT has the potential to eradicate micrometastases, facilitate local control by surgical resection, increase radiosensitivity and decrease the hypoxic cell fraction. However, studies have shown that NACT followed by hysterectomy does not provide any long-term survival benefits for LACC patients when compared to CCRT or early-stage patients who undergo direct surgery^5,6^. Gupta et al. conducted a phase III clinical trial comparing NACT followed by surgery to CCRT in patients with LACC. The study found that NACT followed by surgery did not provide a 5-year disease-free survival (DFS) benefit compared to CCRT, particularly in stage IIB disease. However, the 5-year overall survival (OS) rates were similar between the two groups. Notably, NACT followed by surgery resulted in fewer delayed rectal, bladder, and vaginal toxicities^5^. Despite the lack of evidence, NACT followed by surgery is still being practiced in many parts of the world, particularly in developing countries. Therefore, further research is needed to explore the prognostic factors for CC patients who have undergone NACT to adjust treatment regimens accordingly.

Lymph node metastasis (LNM) is considered a deleterious prognostic indicator for CC and has been incorporated into the 2018 International Federation of Gynecology and Obstetrics (FIGO) stage^7^. The status of lymph nodes can be evaluated either radiologically, through CT, MRI or PET/CT, or pathologically, through lymph node biopsy or post-surgery specimens^8^. Pathological information is used to upgrade or downgrade the final stage of CC and to help establish the post-surgery treatment plan. Studies have reported that NACT decreases LNM^9^. However, the prognostic role of pretreatment radiological LN status (LNr), the response of lymph nodes to NACT, and post-operative pathological lymph node status (LNp) in CC patients who underwent NACT and followed surgery is not yet clear.

Hence, the aim of our study was to investigate the prognostic factors for CC patients who underwent NACT and surgery, with a particular emphasis on the alteration in lymph node status for predicting progression-free survival (PFS) and OS.

## Materials and methods

### Patients

**Figure 1:**
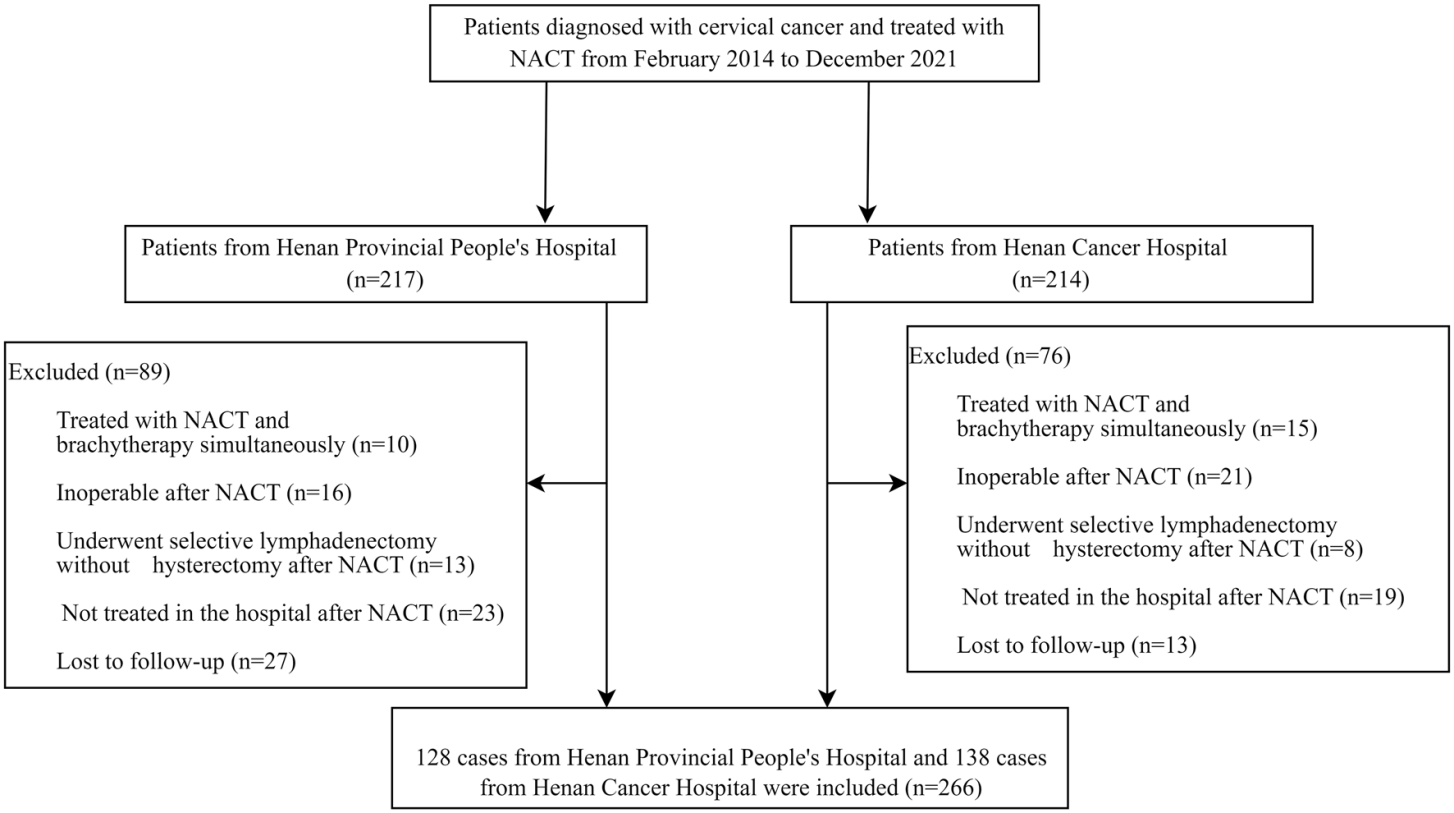
Patient recruitment flowchart**.**

CC patients who underwent radical hysterectomy and lymphadenectomy after NACT between February 2014 and December 2021 were screened for eligibility. This retrospective study was approved by the ethics committee of the Hospital, and informed consent was waived. The inclusion criteria consisted of: (i) patients who underwent pelvic MRI for pretreatment evaluation; (ii) patients who received 1 to 3 cycles of NACT followed by hysterectomy, lymphadenectomy, and detailed pathological evaluation; and (iii) patients who were followed until the date of relapse or death, or until the last follow-up date (October 1, 2022). The exclusion criteria were as follows: (i) patients who underwent NACT with brachytherapy simultaneously; (ii) patients who were deemed inoperable after NACT and subsequently underwent CCRT; (iii) patients who underwent selective lymphadenectomy without hysterectomy after NACT; (iv) patients who were not treated in either hospital after NACT; and (v) patients who were lost to follow-up. The patient recruitment flowchart was shown in Fig. 1.

Clinicopathological characteristics of patients, such as age, FIGO stage, NACT regimen, surgery, and postoperative adjuvant chemotherapy and radiation were retrieved from their medical records.

### Radiological evaluation

The MRI protocols for pelvic imaging are detailed in Table S1.

Two radiologists (Jianghua Lou and Qingxia Wu, with 12 and 13 years of pelvic imaging experience respectively) evaluated the image findings separately on a dedicated Carestream software.

**Figure 2:**
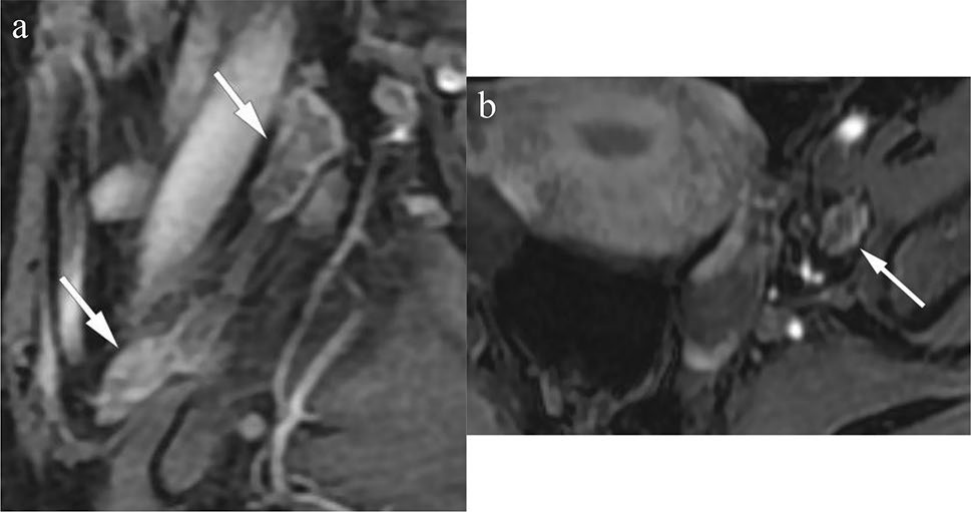
Baseline lymph node measurement and signal intensity evaluation after contrast administration. (**a**) sagittal contrast-enhanced T1WI shows clusters of lymph nodes in the obstrutor area (arrows), the cranial-caudal and antero-posterior diameter of the largest lymph node was measured as 25.8 and 13.3mm (upper arrow). Central necrosis was identified in it. (**b**) Axial contrast-enhanced T1WI shows the transverse diameter of the largest obstrutor lymph node is 9.36mm (arrow). This patient was identified as with positive radiological lymph node.

The baseline tumor size was determined as the maximum tumor diameter measured on the pretreatment MRI. The three dimensions of the lymph node with the largest short diameter were measured, and the signal intensity after Gadolinium administration was assessed, as shown in Fig. 2. The lymph node was considered radiologically positive if it met one of the following criteria: (1) a shortest diameter of more than 8 mm^10^; (2) ring enhancement showing necrosis inside the lymph node.

### Treatment

The NACT protocol included 1 to 3 cycles of platinum-based intravenous chemotherapy administered at three-week intervals. Gynecologists evaluated the feasibility of surgical resection and performed radical hysterectomy and lymphadenectomy 3 weeks after completion of NACT. If pelvic node disease was detected during intraoperative examination or if bulky aortic nodes were identified on preoperative imaging or during surgery, aortic lymphadenectomy was performed.

Tumor response to NACT was evaluated according to response evaluation criteria in solid tumors (RECIST) v1.1^11^. The baseline size of the tumor, as assessed by pretreatment MRI, and the gross morphological tumor size, evaluated by pathologists, were employed for the assessment of short-term response to NACT. Patients who exhibited complete or partial response were designated as responders, while those with stable or progressive disease were classified as non-responders.

### Pathological evaluation

All the resected surgical samples including the uterus and lymph nodes were formalin fixed, paraffin embedded, and then histopathologically diagnosed by the Department of Pathology of the two hospitals. Pathological information, including gross morphological tumor size, histological type, differentiation, stromal invasion depth, lymphovascular space invasion (LVSI), perineural invasion, involvement of uterus corpus and vagina, parametrial infiltration, incisal margin status and lymph node status were retracted from the pathological reports of the patients. Tumor size after NACT was defined as the maximal diameter of the tumor in the resected specimen. Pathological lymph node status, no metastasis [LNp(-)] and LNM [LNp( +)]) were ascertained based on the pathological reports. Both the radiological and pathological lymph node status were used to adjust FIGO stage of the patients to conform with the 2018 staging system^7^. Patients with either LNr( +) or LNp( +) were classified as IIIC no matter what the original FIGO stage was.

### Adjuvant therapy

LNM, parametrial infiltration, and positive incisal margin were considered high-risk factors, while LVSI, stromal invasion, and tumor size were considered intermediate-risk factors^12^. According to the final pathological results, Nearly all patients received four courses of adjuvant chemotherapy, except for those who were unable to tolerate it. Patients with one or more high-risk factors or at least two intermediate-risk factors received adjuvant CCRT^3^. The postoperative chemotherapy regimen comprised intravenous chemotherapy based on platinum as well. Adjuvant radiotherapy encompassed the entire pelvis, incorporating the common iliac and periaortic lymph nodes if they were positive.

### Follow up

Following treatment, patients underwent monitoring at 3-month intervals for the first 2 years, at 4–6 months intervals for the following 2 years, and annually thereafter. Patients were meticulously followed up to identify persistent or recurrent disease through clinical examination and imaging.

The primary and secondary outcome measures in this study were PFS and OS, respectively. PFS was defined as the period from the time of diagnosis to either relapse, the last follow-up date, or any signs of disease progression. OS was defined as the time from diagnosis to either relapse, death, or the last follow-up date^13^.

### Statistical analysis

Data analysis was conducted using SPSS 27.0 and R software (version 3.6.1). The Kolmogorov–Smirnov test was utilized to determine the normality of continuous variables distribution. To investigate the differences between patients with different lymph node status, Student’s t-test, nonparametric Mann–Whitney U test and Pearson Chi-square test were applied. To predict post-NACT LNp, a receiver operating characteristic (ROC) curve was established for baseline LNr. The log-rank test was used to compare survival between patient groups. Cox proportional hazards regression models were employed for both univariate and multivariate analyses to estimate the hazard ratio (HR) of various factors with PFS and OS. Time-dependent ROC curves were performed to evaluate the multivariate models established for PFS and OS. Statistical significance was considered with a 2-sided P ≤ 0.05.

### Ethics approval and consent to participate

This study was performed in accordance with Declaration of Helsinki. This retrospective study was approved by the ethics committee of the Henan Provincial People’s Hospital (HPPH). The informed consent from patients was waived by the ethics committee of the HPPH.

## Results

### Patient characteristics

A total of 266 patients with CC who received NACT followed by hysterectomy and lymphadenectomy were included in this study, with 128 patients from HPPH and 138 from Henan Cancer Hospital (HCH). Of these patients, 78 (29.3%) were diagnosed with LNr( +) before treatment, with 76 (29.1%) having a shortest diameter greater than 8mm and 38 (14.2%) showing necrosis after enhancement. After surgery, 57 (21.4%) patients were found to have positive lymph nodes based on pathological evaluation. The clinicopathological characteristics of all patients and different lymph node status groups are presented in Table 1, while the baseline characteristics of patients in each hospital are provided in Supplementary Tables S2 and S3.

**Table 1:** Clinicopathological characteristics of patients in the entire cohort and in different lymph node status groups.

Characteristics, n (%)	All cohorts (n = 266)	LNr (−) (n = 188)	LNr ( +) (n = 78)	P value	LNp (−) (n = 209)	LNp ( +) (n = 57)	P value
Age							
≤ 50	92 (34.6%)	69 (25.9%)	23 (8.6%)	0.260	71 (26.7%)	21 (7.9%)	0.686
> 50	174 (65.4%)	119 (44.7%)	55 (20.7%)		138 (51.9%)	36 (13.5%)	
NACT cycle							
1	48 (18.0%)	36 (13.5%)	12 (4.5%)	0.650	37 (13.9%)	11 (4.1%)	0.793
2	183 (68.8%)	129 (48.5%)	54 (20.3%)		143 (53.8%)	40 (15%)	
3	35 (13.2%)	23 (8.6%)	12 (4.5%)		29 (10.9%)	6 (2.3%)	
Baseline tumor size (cm)							
≤ 2	7 (2.6%)	7 (2.6%)	0 (0%)	< 0.001	7 (2.6%)	0 (0%)	0.011
> 2, ≤ 4	72 (27.0%)	65 (24.4%)	7 (2.6%)		64 (24.1%)	8 (3%)	
> 4	187 (70.4%)	116 (43.7%)	71 (26.7%)		138 (51.9%)	49 (18.4%)	
Tumor size after NACT (cm)							
≤ 2	95 (35.7%)	75 (28.2%)	20 (7.5%)	0.006	85 (32%)	10 (3.8%)	< 0.001
> 2, ≤ 4	116 (43.6%)	83 (31.2%)	33 (12.4%)		92 (34.6%)	24 (9%)	
> 4	55 (20.7%)	30 (11.3%)	25 (9.4%)		32 (12%)	23 (8.6%)	
Response to NACT							
Responders	160 (60.2%)	118 (44.4%)	42 (15.8%)	0.176	134 (50.4%)	26 (9.8%)	0.011
Non-responders	106 (39.8%)	70 (26.3%)	36 (13.5%)		75 (28.2%)	31 (11.7%)	
FIGO stage							
IB	45 (16.9%)	45 (16.9%)	0 (0%)	< 0.001	45 (16.9%)	0 (0%)	< 0.001
IIA	69 (25.9%)	69 (25.9%)	0 (0%)		69 (25.9%)	0 (0%)	
IIB	37 (13.9%)	37 (13.9%)	0 (0%)		37 (13.9%)	0 (0%)	
IIIC	115 (43.2%)	37 (13.9%)	78 (29.3%)		58 (21.8%)	57 (21.4%)	
Tumor type							
SCC	229 (86.1%)	157 (59%)	72 (27.1%)	0.059	179 (67.3%)	50 (18.8%)	0.684
Non-SCC	37 (13.9%)	31 (11.7%)	6 (2.3%)		30 (11.3%)	7 (2.6%)	
Differentiation							
Well and moderately	198 (74.4%)	144 (54.1%)	54 (20.3%)	0.210	163 (61.3%)	35 (13.2%)	0.011
Poorly	68 (25.5%)	44 (16.5%)	24 (9%)		46 (17.3%)	22 (8.3%)	
Stromal invasion depth							
≤ 1/3	62 (23.3%)	49 (18.4%)	13 (4.9%)	0.009	61 (22.9%)	1 (0.4%)	< 0.001
> 1/3, ≤ 2/3	108 (40.6%)	82 (30.8%)	26 (9.8%)		91 (34.2%)	17 (6.4%)	
> 2/3	96 (26.1%)	57 (21.4%)	39 (14.7%)		57 (21.4%)	39 (14.7%)	
LVSI							
No	168 (63.2%)	131 (49.2%)	37 (13.9%)	< 0.001	160 (60.2%)	8 (3%)	< 0.001
Yes	98 (36.8%)	57 (21.4%)	41 (15.4%)		49 (18.4%)	49 (18.4%)	
Perineural invasion							
No	241 (90.6%)	174 (65.4%)	67 (25.2%)	0.090	196 (73.7%)	45 (16.9%)	< 0.001
Yes	25 (9.4%)	14 (5.3%)	11 (4.1%)		13 (4.9%)	12 (4.5%)	
Corpus involvement							
No	177 (66.5%)	131 (49.2%)	46 (17.3%)	0.092	146 (54.9%)	31 (11.7%)	0.028
Yes	89 (33.5%)	57 (21.4%)	32 (12%)		63 (23.7%)	26 (9.8%)	
Vaginal involvement							
No	239 (89.8%)	170 (63.9%)	69 (25.9%)	0.629	189 (71.1%)	50 (18.8%)	0.548
Yes	27 (10.2%)	18 (6.8%)	9 (3.4%)		20 (7.5%)	7 (2.6%)	
Incision margin							
No	257 (96.6%)	182 (68.4%)	75 (28.2%)	1.000	205 (77.1%)	52 (19.5%)	0.034
Yes	9 (3.4%)	6 (2.3%)	3 (1.1%)		4 (1.5%)	5 (1.9%)	
Parametrial involvement							
No	257 (96.6%)	184 (69.2%)	73 (27.4%)	0.166	206 (77.4%)	51 (19.2%)	0.003
Yes	9 (3.4%)	4 (1.5%)	5 (1.9%)		3 (1.1%)	6 (2.3%)	
Adjuvant radiation							
No	122 (45.9%)	95 (35.7%)	27 (10.2%)	0.018	112 (42.1%)	10 (3.8%)	< 0.001
Yes	144 (54.1%)	93 (35%)	51 (19.2%)		97 (36.5%)	47 (17.7%)	
Adjuvant chemotherapy							
No	6 (2.3%)	4 (1.5%)	2 (0.8%)	1.000	5 (1.9%)	1 (0.4%)	1.000
Yes	260 (97.7%)	184 (69.2%)	76 (28.6%)		204 (76.7%)	56 (21.1%)	

*LNr (−)* negative radiological lymph node, *LNr ( +)* positive radiological lymph node, *LNp (−)* negative pathological lymph node, *LNp ( +)* positive pathological lymph node, *NACT* neoadjuvant chemotherapy, *FIGO* The International Federation of Gynecology and Obstetrics, *SCC* squamous cell carcinoma, *LVSI* lymph-vascular space invasion.

The overall response rate was 60.2% (160/266) based on RECIST v1.1 criteria. The response rate in patients with LNp(-) was 64.1% (134/209), significantly higher than that of 45.6% (26/57) in patients with LNp( +) (p = 0.011) (Table 1).

**Figure 3:**
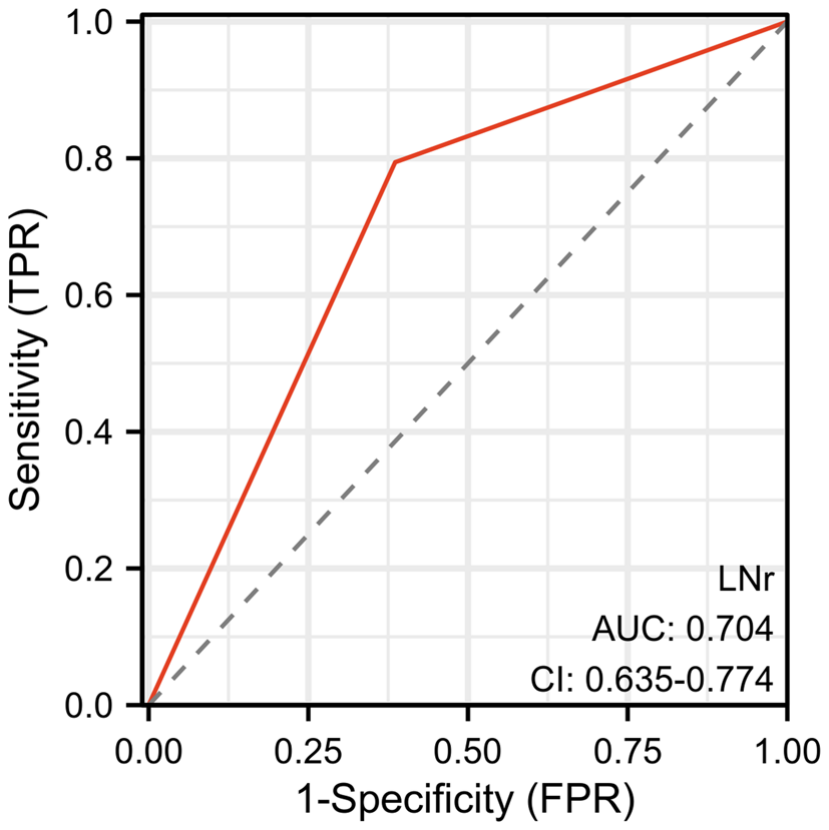
ROC curve of baseline radiological lymph node status for the prediction of pathological lymph node status after NACT. The AUC was 0.704 (95% CI 0.6635–0.774), with sensitivity of 79.4% and specificity of 61.4% respectively.

The LNr assessed at baseline by measuring the shortest diameter and presence of necrosis displayed promising potential for predicting the post-operative LNp.The ROC curve for the baseline LNr in predicting post-operative LNp is displayed in Fig. 3, and the area under ROC curve (AUC) is 0.704 with a 95% confidence interval (CI) of 0.635–0.774.

### Prognostic factors and survival

**Figure 4:**
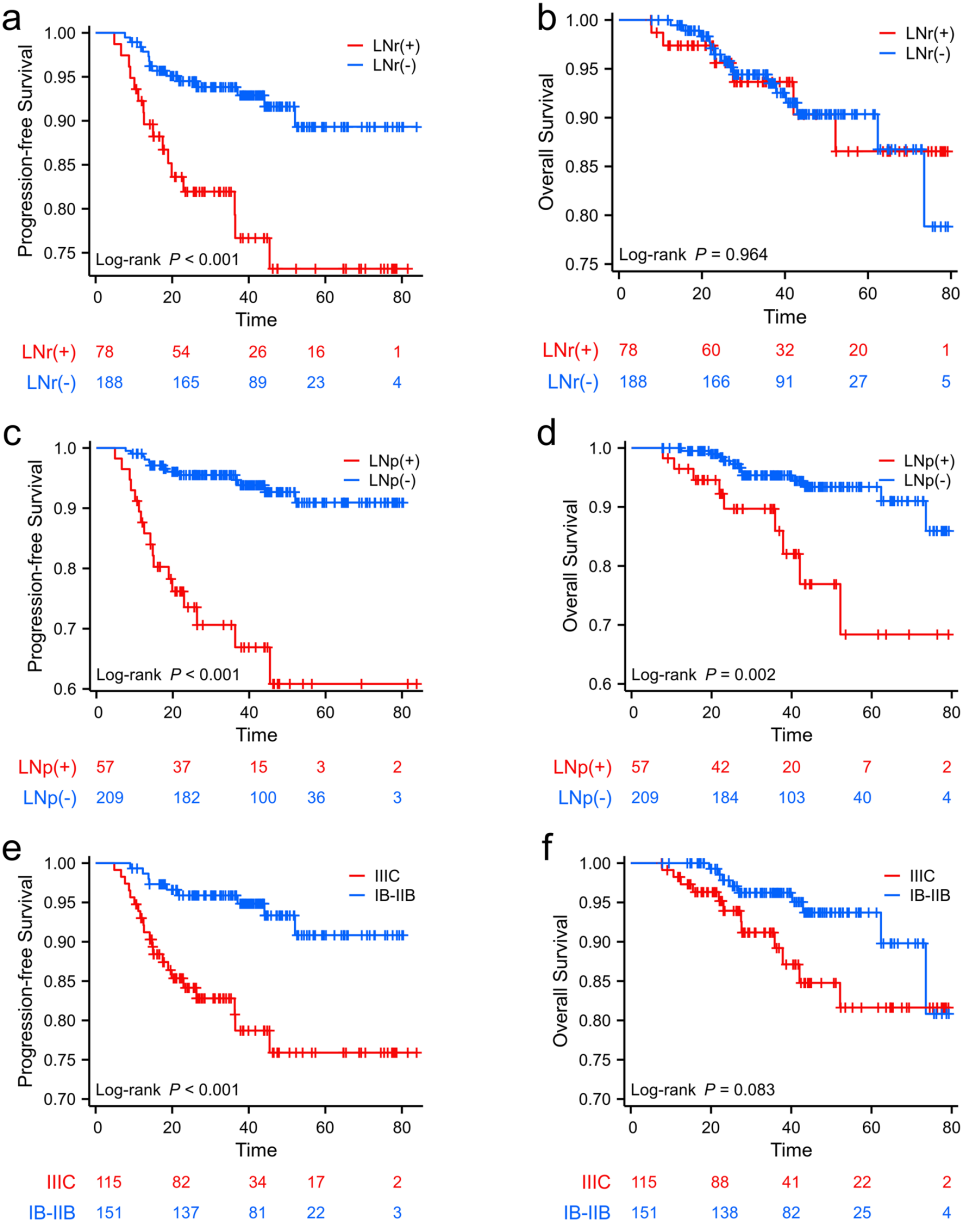
Kaplan–Meier survival analysis of progression-free survival (PFS) and overall survival (OS) according to baseline radiological and post-operative pathological lymph node status and 2018 FIGO stage. Patients with LNr ( +), LNp ( +) and 2018 FIGO IIIC showed worse PFS (**a**,**c**,**e**), whereas for OS the difference was only found in patients with different pathological lymph node status (**b**,**d**,**f**).

The median follow-up period was 36.4 months (range, 4.9 to 83.8 months). By October 2022, 11.2% (30/266) patients experienced disease progression and 7.8% (21/266) patients deceased. The 1, 2 and 3-year PFS rates were 94.9%, 89.4% and 88.0% respectively. The 1, 2 and 3-year OS rates were 97.7%, 96.1% and 92.8% respectively (Table 2). Patients with LNr(-) and LNp(-) showed better 1, 2 and 3-year PFS rates than patients with LNr( +) and LNp( +), with p value of 0.052, 0.021 and 0.019 respectively (Table 2). Whereas the 1, 2 and 3-year OS rates between the two groups were not significantly different. As shown in Fig. 4, the PFS for patients with 2018 FIGO IIIC was significantly lower than that of FIGO IB-IIB (p < 0.001). The 3-year OS for patients with 2018 FIGO IIIC did not differ from patients with stage IB-IIB (p = 0.083).

**Table 2:** 1, 2 and 3-year survival probability (95% CI) in the entire cohort and in different radiological and pathological lymph node status.

	All cohorts	LNr(-)	LNr( +)	P-value	LNp(-)	LNp( +)	P-value
1-year PFS (%)	94.9 ± 1.9	97.9 ± 1.1	90.9 ± 3.2	0.052	98.6 ± 0.8	87.7 ± 4.3	0.020
2-year PFS (%)	89.4 ± 2.7	93.8 ± 1.8	82.0 ± 4.5	0.021	95.5 ± 1.5	73.6 ± 6.2	< 0.001
3-year PFS (%)	88.0 ± 3.0	92.9 ± 2.0	79.3 ± 5.1	0.019	94.7 ± 1.7	66.9 ± 7.2	< 0.001
1-year OS (%)	97.7 ± 1.3	99.5 ± 0.5	97.4 ± 1.8	0.221	99.5 ± 0.5	96.5 ± 2.4	0.191
2-year OS (%)	96.1 ± 1.7	95.8 ± 1.5	95.6 ± 2.5	0.398	97.3 ± 1.2	89.7 ± 4.4	0.057
3-year OS (%)	92.8 ± 2.5	93.5 ± 2.0	90.3 ± 3.1	0.369	94.4 ± 1.8	86.0 ± 5.6	0.142

*PFS* progression-free survival, *OS* overall survival, *LNr (−)* negative radiological lymph node, *LNr ( +)* positive radiological lymph node, *LNp(−)* negative pathological lymph node, *LNp ( +)* positive pathological lymph node.

As shown in Table 3, multivariate analysis identified the main independent predictors of PFS as LNp( +) (HR = 3.777; 95% CI 1.715–8.319), non-SCC (HR = 2.956; 95% CI 1.297–6.736), poor differentiation (HR = 2.370; 95% CI 1.130–4.970) and adjuvant radiation (HR = 3.266; 95% CI 1.183–9.019). As for OS, multivariate analysis identified the main independent predictors of OS as non-SCC (HR = 5.691; 95% CI 2.344–13.815) and LVSI (HR = 5.652; 95% CI 2.227–14.345) (Table 4).

**Table 3:** Univariate and multivariate analysis for progression-free survival in the entire cohort.

Characteristics	Univariate analysis			Multivariate analysis		
	HR	95% CI	P value	HR	95% CI	P value
Age (≤ 50 vs. > 50)	0.820	0.394–1.705	0.597			
NACT cycle (≤ 2 vs. > 2)	1.032	0.360–2.956	0.954			
Baseline tumor size (≤ 4 vs. > 4)	3.010	1.050–8.633	0.040			
Tumor size after NACT (≤ 4 vs. > 4)	1.930	0.881–4.227	0.100			
Response to NACT (No vs. Yes)	0.537	0.262–1.100	0.089			
FIGO stage (IB-IIB vs. IIIC)	3.739	1.708–8.186	< 0.001			
Tumor type (SCC vs. Non-SCC)	2.487	1.106–5.593	0.028	2.956	1.297–6.736	0.010
Differentiation (Well and moderately vs. Poorly)	3.249	1.587–6.654	0.001	2.370	1.130–4.970	0.022
LVSI (No vs. Yes)	4.525	2.101–9.744	< 0.001			
Stromal invasion depth (≤ 2/3 vs. > 2/3)	3.175	1.526–6.603	0.002			
Perineural invasion (No vs. Yes)	1.875	0.651–5.398	0.244			
Corpus involvement (No vs. Yes)	0.875	0.400–1.911	0.737			
Vaginal involvement (No vs. Yes)	1.635	0.569–4.692	0.361			
Incision margin (No vs. Yes)	3.966	1.198–13.125	0.024			
Parametrial involvement (No vs. Yes)	4.701	1.638–13.491	0.004			
Adjuvant radiation (No vs. Yes)	4.882	1.867–12.766	0.001	3.266	1.183–9.019	0.022
Adjuvant chemotherapy (No vs. Yes)	0.696	0.095–5.116	0.722			
LNr (No vs. Yes)	3.154	1.538–6.469	0.002			
LNp (No vs. Yes)	6.226	3.009–12.882	< 0.001	3.777	1.715–8.319	< 0.001

*HR* hazard ratio, *CI* confidence interval, *NACT* neoadjuvant chemotherapy, *FIGO* The International Federation of Gynecology and Obstetrics, *SCC* squamous cell carcinoma, *LVSI* lymph-vascular space invasion, *LNr* radiological lymph node status, *LNp* pathological lymph node status.

**Table 4:** Univariate and multivariate analysis for overall survival in the entire cohort.

Characteristics	Univariate analysis			Multivariate analysis		
	HR	95% CI	P value	HR	95% CI	P value
Age (≤ 50 vs. > 50)	1.429	0.552–3.697	0.462			
NACT cycle (≤ 2 vs. > 2)	1.083	0.318–3.686	0.898			
Baseline tumor size (≤ 4 vs. > 4)	1.417	0.519–3.873	0.496			
Tumor size after NACT (≤ 4 vs. > 4)	1.128	0.378–3.371	0.829			
Response to NACT (No vs. Yes)	0.471	0.198–1.118	0.088			
FIGO stage (IB-IIB vs. IIIC)	2.121	0.890–5.056	0.090			
Tumor type (SCC vs. Non-SCC)	5.118	2.144–12.219	< 0.001	5.691	2.344–13.815	< 0.001
Differentiation (Well and moderately vs. Poorly)	2.098	0.881–4.995	0.094			
LVSI (No vs. Yes)	5.150	2.062–12.863	< 0.001	5.652	2.227–14.345	< 0.001
Stromal invasion depth (≤ 2/3 vs. > 2/3)	3.076	1.293–7.317	0.011			
Perineural invasion (No vs. Yes)	4.272	1.404–12.997	0.011			
Corpus involvement (No vs. Yes)	1.643	0.692–3.901	0.261			
Vaginal involvement (No vs. Yes)	1.940	0.569–6.610	0.290			
Incision margin (No vs. Yes)	4.103	0.951–17.704	0.058			
Parametrial involvement (No vs. Yes)	4.406	1.295–14.986	0.018			
Adjuvant radiation (No vs. Yes)	1.984	0.800–4.921	0.139			
Adjuvant chemotherapy (No vs. Yes)	20.951	0–3088071	0.616			
LNr (No vs. Yes)	0.978	0.376–2.546	0.964			
LNp (No vs. Yes)	3.559	1.496–8.469	0.004			

*HR* hazard ratio, *CI* confidence interval, *NACT* neoadjuvant chemotherapy, *FIGO* The International Federation of Gynecology and Obstetrics, *SCC* squamous cell carcinoma, *LVSI* lymph-vascular space invasion, *LNr* radiological lymph node status, *LNp* pathological lymph node status.

**Figure 5:**
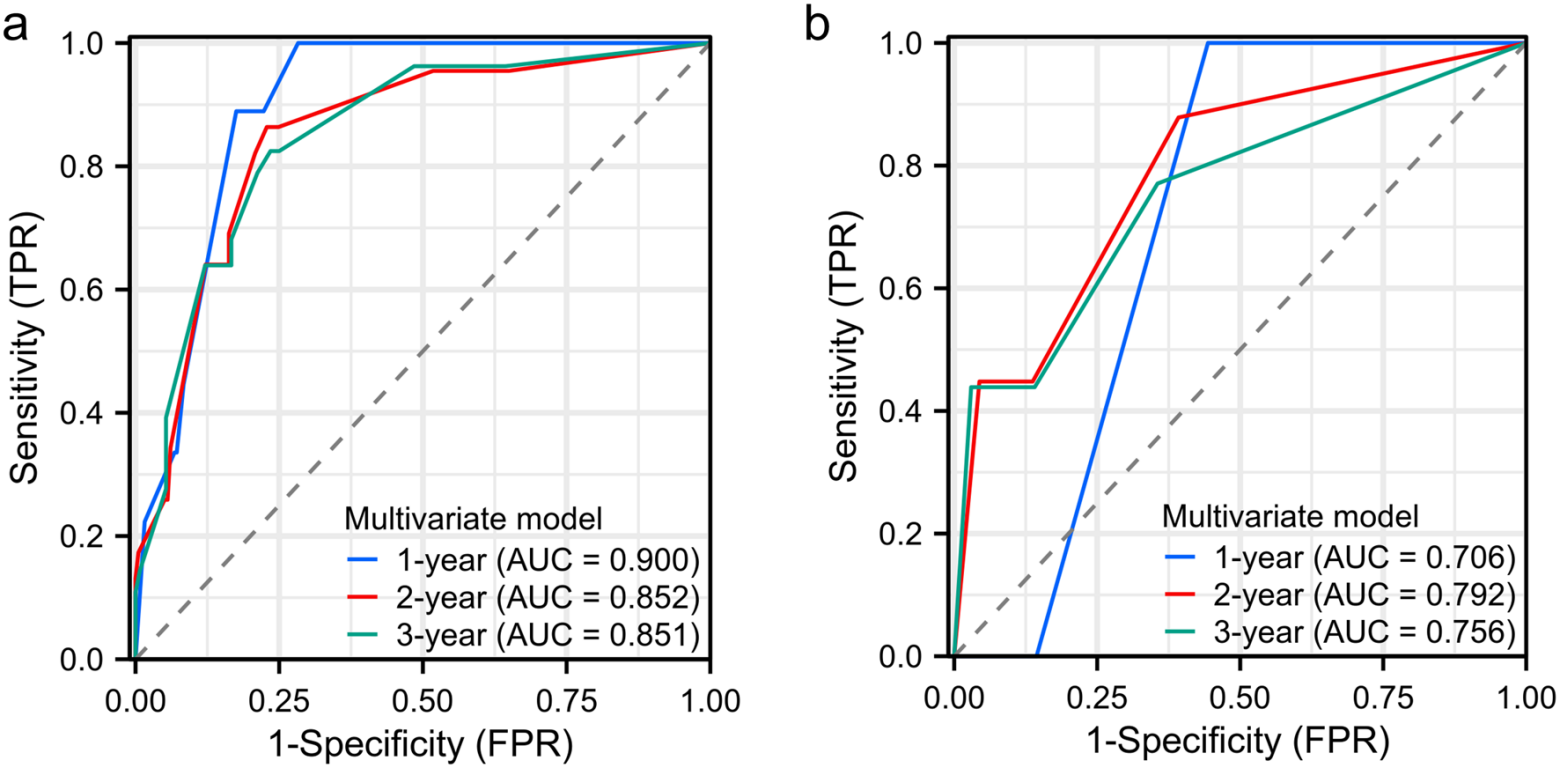
The 1,2 and 3-year time-dependent ROC curves of the multivariate model for PFS and OS. The AUCs were 0.900, 0.852, and 0.851 for PFS, and 0.706, 0.792, and 0.756 for OS, respectively.

The 1,2 and 3-year time-dependent ROC curves of the multivariate model for PFS and OS were shown in Fig. 5. The AUCs were 0.900, 0.852, and 0.851 for PFS, and 0.706, 0.792, and 0.756 for OS, respectively.

### The interaction between LNr and LNp

There was an interaction between LNr and LNp regarding PFS (P = 1.5357e − 7 for univariate analysis and P = 0.000171 for multivariate analysis), whereas the interaction did not exist regarding OS (P = 0.063 for univariate analysis and P = 0.06681 for multivariate analysis).

**Figure 6:**
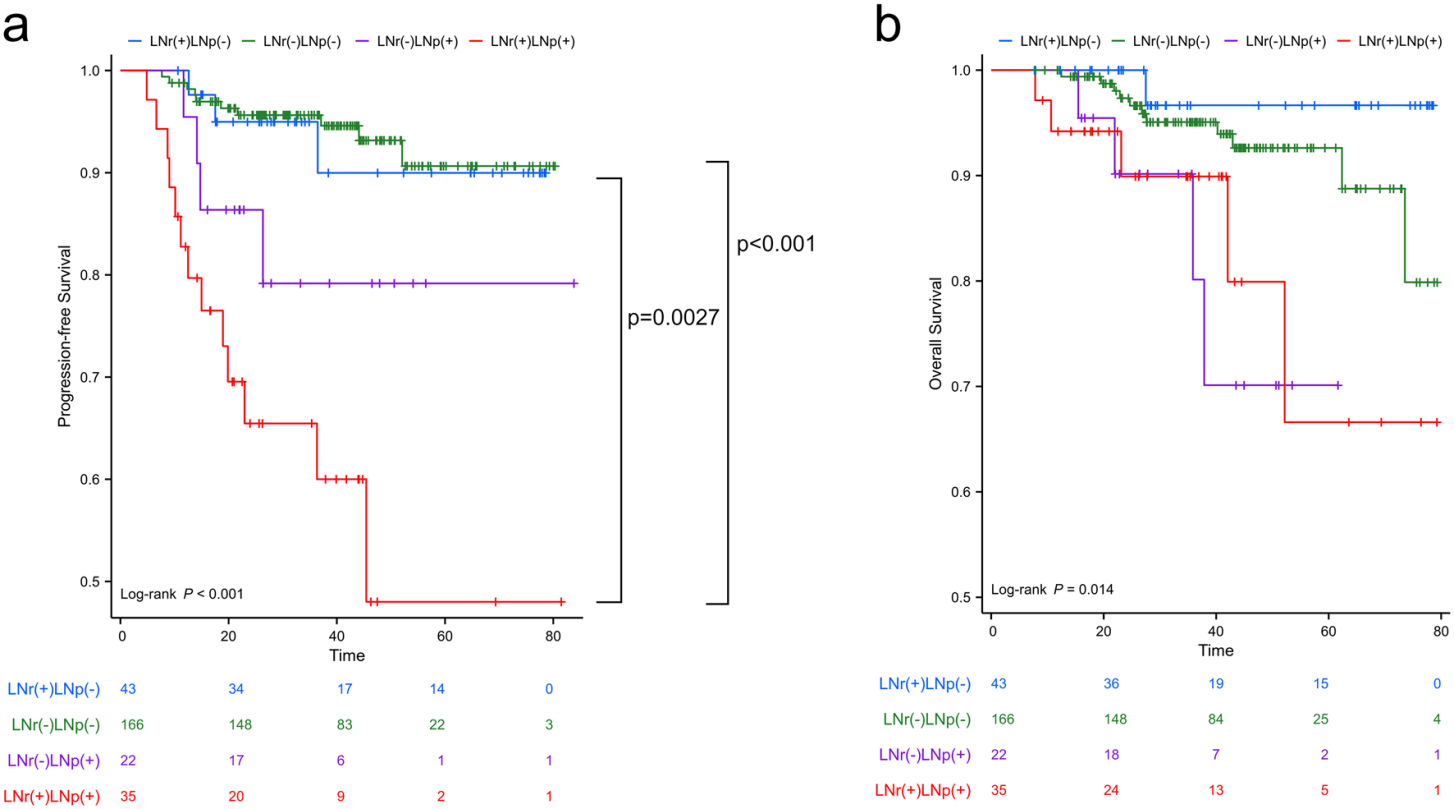
Kaplan–Meier survival analysis of progression-free survival (PFS) and overall survival (OS) stratified by combined radiological and pathological lymph node status. Patients with concurrent LNr ( +) and LNp ( +) demonstrated the worst PFS, and patients with both LNr (−) and LNp (−) showed the best PFS (**a**). In patients with LNr ( +), the pairwise comparison indicated a significant difference in PFS between patients with LNp (−) and LNp ( +) (p = 0.0027). There were no statistical differences observed in the pairwise comparison between subgroups concerning OS (**b**).

After stratification into four mutually exclusive subgroups based on the combined LNr and LNp, patients with concurrent LNr( +) and LNp( +) demonstrated the worst PFS, and patients with both LNr(−) and LNp(−) showed the best PFS, as depicted in Fig. 6. In patients with LNr( +), the pairwise comparison indicated a significant difference in PFS between patients with LNp(−) and LNp( +) (p = 0.0027), while in patients with LNr(−), there was no significant difference in PFS between patients with LNp(−) and LNp( +) (p = 0.154). Notably, the adverse prognostic impact of LNp( +) was more pronounced in patients with LNr( +).

## Discussion

This retrospective, observational and multicenter study demonstrates that in CC patients who underwent NACT and followed hysterectomy, patients with FIGO 2018 stage IIIC had worse PFS compared to those with IB-IIB. LNp( +), non-SCC, poor differentiation and adjuvant radiation were identified as independent risk factors for PFS. Our findings suggest that the adverse prognostic value of LNp( +) was more significant in patients with LNr( +), highlighting the importance of accurate lymph node staging before treatment.

CC has moderate sensitivity to chemotherapy, with clinical short-term response rates ranging from 30 to 90%^14–16^. In our study, the response rate in patients with LNp(-) was 64.1%, significantly higher than that of 45.6% in patients with LNp( +). It is believed that patients who respond to NACT will benefit from this treatment^16^ and studies have been conducted to predict responders in order to obtain survival benefits^17^. However, both our present study and a randomized clinical trial showed that response to NACT does not necessarily mean favorable survival outcomes^15^. The inconsistent correlation between clinical response to NACT and long-term survival implies that future studies should focus on identifying more robust prognostic factors, such as positive lymph node and LVSI after NACT, which were identified as independent risk factors for PFS and OS in our study.

A meta-analysis found that the short-term outcomes of non-SCC were similar to SCC, however, patients with SCC experienced a significant better 5-year OS and PFS when compared to patients with non-SCC^18–20^. This is also found in our study. Non-SCC and tumors with poor differentiation showed a higher risk for PFS, both tumor type and differentiation are independent risk factors for PFS. To date, the recommended management of CC is merely guided by FIGO staging at diagnosis^3^. This highlights the need for specific therapeutic strategies based on molecular characterization to identify targets and develop novel treatments.

Our study demonstrated a significant difference in perineural invasion, corpus and parametrial involvement, and positive incision margin between patients with LNp(−) and LNp( +), while not in patients with LNr(−) and LNr( +). We believe that this can be attributed to two factors. Firstly, patients with LNp( +) after NACT are indicative of those with more advanced disease and poor response to NACT. Prior studies have indicated that CC patients with LNM exhibit higher rates of parametrial invasion, LVSI, and perineural invasion^21–23^. Secondly, this is due to the discrepancy between LNr status and LNp status, as LNr status does not fully reflect true LNM.

Since the implementation of FIGO 2018 staging, many studies have been conducted to evaluate the prognosis value of stage IIIC and found that survival of stage IIIC varied significantly and differed based on T stages^24,25^. Our study revealed that patients with stage IIIC had a lower PFS compared to those with stages IB-IIB. We identified that a post-NACT LNp( +) is an independent determinant for PFS, but not for OS. Patients with LNp( +) are recommended to undergo adjuvant radiation therapy. The exclusion of this factor from the multivariate model for OS in our study may suggest that the survival disadvantage associated with LNM could potentially be counteracted by adjuvant radiation therapy.

Pinto et al. reported that the size of pretreatment pelvic lymph nodes was negatively associated with OS in patients with 2018 FIGO IIIC1^26^. Our study also observed a similar finding. As expected, patients with LNr( +) LNp( +) had the worst survival, while those with LNr(-) LNp(-) had the best survival. For the intermediate cohort of LNr( +) LNp(−) and LNr(−) LNp( +), patients with LNr( +) LNp(−) had a better survival compared to those with LNr(−) LNp( +), although this difference was not statistically significant. The results suggest that even patients with LNp( +) that cannot be identified radiologically represent a relatively worse subset compared to those with LNr( +), who may respond better to NACT. This suggests that, on one hand, post-NACT LNp status is more important for predicting PFS than baseline LNr status, and on the other hand, the lymph node response to NACT may also contribute to better survival outcomes. Further comparisons between the lymph node groups suggest that the adverse prognostic value of LNp( +) was more significant in patients with LNr( +).

Our study is the first to establish risk stratification for survival based on pre- and post-treatment lymph node status. However, there are several limitations. First, it is a retrospective study with a relatively small number of events. There were less than 5 events of PFS in the subgroups of LNr( +) LNp(−) and LNr(−) LNp( +), despite the data being collected from two tertiary centers. Second, post-NACT imaging was lacking for tumor extent and lymph node evaluation in our study, and the possibility of surgical resection was mainly based on clinical examination in the two hospitals. A change in lymph node evaluation based on imaging might better reflect its response to NACT. Third, due to the relatively small number of patients with DFS events, we did not further stratify patients based on FIGO stage. This may have obscured different prognoses among patients.

This study elucidates the prognostic determinants of CC patients who underwent NACT and following surgery, and establishes a risk stratification based on the LN status pre- and post-NACT treatment, which can help guide future treatment. Future research is warranted to determine the risk stratification in subgroups of different tumor histology and FIGO stages. Moreover, larger cohort with more events and independent validation are required to confirm the robustness and generalizability of the findings.

## Conclusion

Pathologically positive lymph node, non-SCC, poor differentiation and adjuvant radiation were independent risk factors for PFS in patients with CC who underwent NACT and followed hysterectomy. The adverse prognostic value of pathologically positive lymph node was more significant in patients with radiologically positive lymph node.

### Supplementary Information

Supplementary Tables.

## Data Availability

The datasets used and/or analysed during the current study are available from the corresponding author on reasonable request.

## Abbreviations

CC: Cervical cancer
LACC: Locally advanced cervical cancer
CCRT: Concurrent chemotherapy and radiotherapy
NACT: Neoadjuvant chemotherapy
LNM: Lymph node metastasis
FIGO: The International Federation of Gynecology and Obstetrics
LNr: Radiological lymph node status
LNp: Pathological lymph node status
PFS: Progression-free survival
OS: Overall survival
HPPH: Henan Provincial People's Hospital
HCH: Henan Cancer Hospital
LVSI: Lymphovascular space invasion
RECIST: Response evaluation criteria in solid tumors
ROC: Receiver operating characteristic
HR: Hazard ratio
AUC: Area under ROC curve
CI: Confidence interval
SCC: Squamous cell carcinoma

## References

[1] Siegel RL, Miller KD, Mbbs NSW, Dvm AJ (2023). Cancer statistics, 2023. CA Cancer J. Clin..

[2] Dostalek L (2018). ESGO survey on current practice in the management of cervical cancer. Int. J. Gynecol. Cancer.

[3] NCCN Guidelines for Patients: Cervical Cancer. *Cerv. Cancer* (2022).

[4] Miriyala R, Mahantshetty U, Maheshwari A, Gupta S (2022). Neoadjuvant chemotherapy followed by surgery in cervical cancer: Past, present and future. Int. J. Gynecol. Cancer.

[5] Gupta S (2018). Neoadjuvant chemotherapy followed by radical surgery versus concomitant chemotherapy and radiotherapy in patients with stage IB2, IIA, or IIB squamous cervical cancer: A randomized controlled trial. J. Clin. Oncol..

[6] Kim HS (2013). Efficacy of neoadjuvant chemotherapy in patients with FIGO stage IB1 to IIA cervical cancer: An international collaborative meta-analysis. Eur. J. Surg. Oncol. EJSO.

[7] Bhatla N (2019). Revised FIGO staging for carcinoma of the cervix uteri. Int. J. Gynecol. Obstet..

[8] Salvo G, Odetto D, Pareja R, Frumovitz M, Ramirez PT (2020). Revised 2018 International Federation of Gynecology and Obstetrics (FIGO) cervical cancer staging: A review of gaps and questions that remain. Int. J. Gynecol. Cancer.

[9] Chen B (2020). The effect of neoadjuvant chemotherapy on lymph node metastasis of FIGO stage IB1-IIB cervical cancer: A systematic review and meta-analysis. Front. Oncol..

[10] Paño B (2015). Pathways of lymphatic spread in gynecologic malignancies. RadioGraphics.

[11] Eisenhauer EA (2009). New response evaluation criteria in solid tumours: Revised RECIST guideline (version 1.1). Eur. J. Cancer.

[12] Sedlis A (1999). A randomized trial of pelvic radiation therapy versus no further therapy in selected patients with stage IB carcinoma of the cervix after radical hysterectomy and pelvic lymphadenectomy: A gynecologic oncology group study. Gynecol. Oncol..

[13] Qian ZR (2018). Association of alterations in main driver genes with outcomes of patients with resected pancreatic ductal adenocarcinoma. JAMA Oncol..

[14] Gong L (2016). Safety and efficacy of neoadjuvant chemotherapy followed by radical surgery versus radical surgery alone in locally advanced cervical cancer patients. Int. J. Gynecol. Cancer.

[15] Hu Y (2022). Neoadjuvant chemotherapy for patients with international federation of gynecology and obstetrics stages IB3 and IIA2 cervical cancer: A multicenter prospective trial. BMC Cancer.

[16] Huang Y (2020). The efficacy and response predictors of platinum-based neoadjuvant chemotherapy in locally advanced cervical cancer. Cancer Manag. Res..

[17] Sun C (2019). Radiomic analysis for pretreatment prediction of response to neoadjuvant chemotherapy in locally advanced cervical cancer: A multicentre study. EBioMedicine.

[18] Couvreur K (2018). Neo-adjuvant treatment of adenocarcinoma and squamous cell carcinoma of the cervix results in significantly different pathological complete response rates. BMC Cancer.

[19] He L (2014). The efficacy of neoadjuvant chemotherapy in different histological types of cervical cancer. Gynecol. Oncol..

[20] Legge F (2022). Locally advanced cervical carcinoma patients treated with chemoradiation followed by radical surgery: Clinical response and oncological outcomes according to histotype after propensity score analysis. Eur. J. Surg. Oncol..

[21] Yang Q (2021). Retrospective analysis of the incidence and predictive factors of parametrial involvement in FIGO IB1 cervical cancer. J. Gynecol. Obstet. Hum. Reprod..

[22] Liu Y, Huang S, Ming X, Jing H, Li Z (2021). Surgical approach and use of uterine manipulator are not associated with LVSI in surgery for early-stage cervical cancer. J. Minim. Invasive Gynecol..

[23] Tang M (2018). Perineural invasion as a prognostic risk factor in patients with early cervical cancer. Oncol. Lett..

[24] Kim J (2021). Magnetic resonance imaging-based validation of the 2018 FIGO staging system in patients treated with definitive radiotherapy for locally advanced cervix cancer. Gynecol. Oncol..

[25] Matsuo K, Machida H, Mandelbaum RS, Konishi I, Mikami M (2019). Validation of the 2018 FIGO cervical cancer staging system. Gynecol. Oncol..

[26] Pinto PJJ (2022). Prognostic factors in locally advanced cervical cancer with pelvic lymph node metastasis. Int. J. Gynecol. Cancer.

