# Supplementary material for: The prognostic value of radiological and pathological lymph node status in patients with cervical cancer who underwent neoadjuvant chemotherapy and followed hysterectomy
Source: Sci Rep. 2024 Jan 24;14:2045. doi: 10.1038/s41598-023-49539-7 (PMC10808453; doi:10.1038/s41598-023-49539-7)
Supplement: Supplementary file 1 — Supplementary Tables. [file 41598_2023_49539_MOESM1_ESM.docx]

Table S1 MRI protocols of the two centers

| **Hospital** | **Scanner** | **Sequence** | **TR (ms)** | **TE (ms)** | **Slice Thickness (mm)** | **Slice Gap (mm)** | **Bandwidth** | **Slices** |
| --- | --- | --- | --- | --- | --- | --- | --- | --- |
| **#1** | GE 3.0T  (Discovery MR 750) | Sagittal T2 | 4000 | 87 | 5 | 1 | 163 | 20 |
|  |  | Coronal T2 | 3927 | 81 | 5 | 1 | 122 | 20 |
|  |  | Axial T2 | 4167 | 87 | 5 | 2 | 139 | 22 |
|  |  | Axial T1 | 446 | 7 | 5 | 2 | 325 | 22 |
|  |  | Axial DWI | 2600 | 74 | 5 | 2 | 1953 | 22 |
|  |  | Axial T1 C+ | 4 | 2 | 2 | 0 | 558 | 108 |
|  |  | Sagittal T1C+ | 4 | 2 | 1.5 | 0 | 558 | 96 |
|  |  | Coronal T1C+ | 4 | 2 | 2 | 0 | 651 | 64 |
|  |  |  |  |  |  |  |  |  |
|  | SIEMENS 3.0T  (TrioTim) | Sagittal T2 | 3000 | 96 | 4 | 1.2 | 250 | 21 |
|  |  | Coronal T2 | 4000 | 104 | 5 | 1 | 203 | 20 |
|  |  | Axial T2 | 4000 | 87 | 5 | 1.5 | 250 | 21 |
|  |  | Axial T1 | 700 | 11 | 5 | 1.5 | 203 | 21 |
|  |  | Axial DWI | 4500 | 76 | 5 | 1.5 | 1736 | 21 |
|  |  | Axial T1 C+ | 3.3 | 1.1 | 1.5 | 0 | 501 | 96 |
|  |  | Sagittal T1C+ | 3.3 | 1.2 | 2 | 0 | 501 | 96 |
|  |  | Coronal T1C+ | 3.3 | 1.1 | 1.5 | 0 | 501 | 96 |
|  |  |  |  |  |  |  |  |  |
| **#2** | GE 1.5T  (Signa HDxt) | Sagittal T2 | 5780 | 133 | 4 | 0.4 | 162.77 | 24 |
|  |  | Axial T2 | 6020 | 128 | 5 | 0.5 | 162.77 | 24 |
|  |  | Axial T1 | 520 | 7 | 5 | 0.5 | 244.14 | 24 |
|  |  | Axial DWI | 2250 | 64 | 5 | 0.5 | 1953.12 | 24 |
|  |  | Axial T1 C+ | 3.7 | 1.5 | 4 | 2 | 488.28 | 68 |
|  |  | Sagittal T1C+ | 3.3 | 1.5 | 2.4 | 1.2 | 325.51 | 88 |
|  |  |  |  |  |  |  |  |  |
|  | SIEMENS  3.0T  (Prisma) | Sagittal T2 | 5000 | 91 | 4 | 0.4 | 200 | 24 |
|  |  | Axial T2 | 4130 | 87 | 5 | 0.5 | 200 | 24 |
|  |  | Axial T1 | 600 | 21 | 5 | 0.5 | 520 | 24 |
|  |  | Axial DWI | 5190 | 59 | 5 | 0.5 | 960 | 24 |
|  |  | Axial T1 C+ | 6.1 | 2.9 | 3 | 0 | 520 | 48 |
|  |  | SagittalT1C+ | 6.3 | 3.0 | 2.5 | 0 | 520 | 44 |

Table S2 Clinicopathological characteristics of patients from Henan Provincial People’s Hospital and in different lymph node status groups

| Characteristics, n (%) | All cohorts (n=128) | LNr(-)  (n=93) | LNr(+)  (n=35) | P value | LNp(-)  (n=99) | LNp(+)  (n=29) | P value |
| --- | --- | --- | --- | --- | --- | --- | --- |
| Age |  |  |  | 0.925 |  |  | 0.107 |
| ≤50 | 54(42.2%) | 39 (30.5%) | 15 (11.7%) |  | 38 (29.7%) | 16 (12.5%) |  |
| >50 | 74(57.8%) | 54 (42.2%) | 20 (15.6%) |  | 61 (47.7%) | 13 (10.2%) |  |
| NACT cycle |  |  |  | 0.336 |  |  | 0.864 |
| 1 | 29(22.7%) | 24 (18.8%) | 5 (3.9%) |  | 22 (17.2%) | 7 (5.5%) |  |
| 2 | 82(64%) | 58 (45.3%) | 24 (18.8%) |  | 63 (49.2%) | 19 (14.8%) |  |
| 3 | 17(13.3%) | 11 (8.6%) | 6 (4.7%) |  | 14 (10.9%) | 3 (2.3%) |  |
| Baseline tumor size (cm) |  |  |  | < 0.001 |  |  | 0.031 |
| ≤2 | 4(3.1%) | 4 (3.1%) | 0 (0%) |  | 4 (3.1%) | 0 (0%) |  |
| >2, ≤4 | 49(38.3%) | 45 (35.2%) | 4 (3.1%) |  | 43 (33.6%) | 6 (4.7%) |  |
| >4 | 75(58.6%) | 44 (34.4%) | 31 (24.2%) |  | 52 (40.6%) | 23 (18%) |  |
| Tumor size after NACT (cm) |  |  |  | 0.061 |  |  | 0.092 |
| ≤2 | 56(43.8%) | 44 (34.4%) | 12 (9.4%) |  | 48 (37.5%) | 8 (6.2%) |  |
| >2, ≤4 | 59(46.1%) | 43 (33.6%) | 16 (12.5%) |  | 43 (33.6%) | 16 (12.5%) |  |
| >4 | 13(10.1%) | 6 (4.7%) | 7 (5.5%) |  | 8 (6.2%) | 5 (3.9%) |  |
| Response to NACT |  |  |  | 0.514 |  |  | 0.257 |
| Responders | 46(35.9%) | 35 (27.3%) | 11 (8.6%) |  | 33 (25.8%) | 13 (10.2%) |  |
| Non-responders | 82(64.1%) | 58 (45.3%) | 24 (18.8%) |  | 66 (51.6%) | 16 (12.5%) |  |
| FIGO stage |  |  |  | < 0.001 |  |  | < 0.001 |
| IB | 15(11.7%) | 15 (11.7%) | 0 (0%) |  | 15 (11.7%) | 0 (0%) |  |
| IIA | 29 (22.7%) | 29 (22.7%) | 0 (0%) |  | 29 (22.7%) | 0 (0%) |  |
| IIB | 31(24.2%) | 31 (24.2%) | 0 (0%) |  | 31 (24.2%) | 0 (0%) |  |
| IIIC | 53(41.4%) | 18 (14.1%) | 35 (27.3%) |  | 24 (18.8%) | 29 (22.7%) |  |
| Tumor type |  |  |  | 0.221 |  |  | 1.000 |
| SCC | 109(85.2%) | 77 (60.2%) | 32 (25%) |  | 84 (65.6%) | 25 (19.5%) |  |
| Non-SCC | 19(14.8%) | 16 (12.5%) | 3 (2.3%) |  | 15 (11.7%) | 4 (3.1%) |  |
| Differentiation |  |  |  | 0.297 |  |  | 0.049 |
| Well and moderately | 106(82.8%) | 79 (61.7%) | 27 (21.1%) |  | 86 (67.2%) | 20 (15.6%) |  |
| Poorly | 22(17.2%) | 14 (10.9%) | 8 (6.2%) |  | 13 (10.2%) | 9 (7%) |  |
| Stromal invasion depth |  |  |  | 0.002 |  |  | 0.001 |
| ≤1/3 | 29(22.7%) | 22 (17.2%) | 7 (5.5%) |  | 28 (21.9%) | 1 (0.8%) |  |
| >1/3, ≤2/3 | 49(38.3%) | 43 (33.6%) | 6 (4.7%) |  | 40 (31.2%) | 9 (7%) |  |
| >2/3 | 50(39%) | 28 (21.9%) | 22 (17.2%) |  | 31 (24.2%) | 19 (14.8%) |  |
| LVSI |  |  |  | 0.002 |  |  | < 0.001 |
| No | 72(56.3%) | 60 (46.9%) | 12 (9.4%) |  | 67 (52.3%) | 5 (3.9%) |  |
| Yes | 56(43.8%) | 33 (25.8%) | 23 (18%) |  | 32 (25%) | 24 (18.8%) |  |
| Perineural invasion |  |  |  | 0.118 |  |  | 0.192 |
| No | 109(85.2%) | 82 (64.1%) | 27 (21.1%) |  | 87 (68%) | 22 (17.2%) |  |
| Yes | 19(14.8%) | 11 (8.6%) | 8 (6.2%) |  | 12 (9.4%) | 7 (5.5%) |  |
| Corpus involvement |  |  |  | 0.038 |  |  | 0.178 |
| No | 84(65.6%) | 66 (51.6%) | 18 (14.1%) |  | 68 (53.1%) | 16 (12.5%) |  |
| Yes | 44(34.4%) | 27 (21.1%) | 17 (13.3%) |  | 31 (24.2%) | 13 (10.2%) |  |
| Vaginal involvement |  |  |  | 1.000 |  |  | 1.000 |
| No | 112(87.5%) | 81 (63.3%) | 31 (24.2%) |  | 87 (68%) | 25 (19.5%) |  |
| Yes | 16(12.5%) | 12 (9.4%) | 4 (3.1%) |  | 12 (9.4%) | 4 (3.1%) |  |
| Incision margin |  |  |  | 1.000 |  |  | 0.396 |
| No | 121(94.5%) | 88 (68.8%) | 33 (25.8%) |  | 95 (74.2%) | 26 (20.3%) |  |
| Yes | 7(5.5%) | 5 (3.9%) | 2 (1.6%) |  | 4 (3.1%) | 3 (2.3%) |  |
| Parametrial involvement |  |  |  | 0.058 |  |  | 0.019 |
| No | 120(93.8%) | 90 (70.3%) | 30 (23.4%) |  | 96 (75%) | 24 (18.8%) |  |
| Yes | 8(6.2%) | 3 (2.3%) | 5 (3.9%) |  | 3 (2.3%) | 5 (3.9%) |  |
| Adjuvant radiation |  |  |  | 0.016 |  |  | 0.003 |
| No | 43(33.6%) | 37 (28.9%) | 6 (4.7%) |  | 40 (31.2%) | 3 (2.3%) |  |
| Yes | 85(66.4%) | 56 (43.8%) | 29 (22.7%) |  | 59 (46.1%) | 26 (20.3%) |  |
| Adjuvant chemotherapy |  |  |  | 1.000 |  |  | 1.000 |
| No | 6(4.7%) | 4 (3.1%) | 2 (1.6%) |  | 5 (3.9%) | 1 (0.8%) |  |
| Yes | 122(95.3%) | 89 (69.5%) | 33 (25.8%) |  | 94 (73.4%) | 28 (21.9%) |  |

Abbreviations: LNr(-), negative radiological lymph node; LNr(+), positive radiological lymph node; LNp(-), negative pathological lymph node; LNp(+), positive pathological lymph node. NACT, neoadjuvant chemotherapy; FIGO, The International Federation of Gynecology and Obstetrics; SCC, squamous cell carcinoma; LVSI, lymph-vascular space invasion.

Table S3 Clinicopathological characteristics of patients from Henan Cancer Hospital and in different lymph node status groups

| Characteristics, n (%) | All cohort (n=138) | LNr(-)  (n=95) | LNr(+)  (n=43) | P value | LNp(-)  (n=110) | LNp(+)  (n=28) | P value |
| --- | --- | --- | --- | --- | --- | --- | --- |
| Age |  |  |  | 0.114 |  |  | 0.199 |
| ≤50 | 38(27.5%) | 30 (21.7%) | 8 (5.8%) |  | 33 (23.9%) | 5 (3.6%) |  |
| >50 | 100(72.5%) | 65 (47.1%) | 35 (25.4%) |  | 77 (55.8%) | 23 (16.7%) |  |
| NACT cycle |  |  |  | 0.808 |  |  | 0.919 |
| 1 | 19(13.8%) | 12 (8.7%) | 7 (5.1%) |  | 15 (10.9%) | 4 (2.9%) |  |
| 2 | 101(73.2%) | 71 (51.4%) | 30 (21.7%) |  | 80 (58%) | 21 (15.2%) |  |
| 3 | 18(13.0%) | 12 (8.7%) | 6 (4.3%) |  | 15 (10.9%) | 3 (2.2%) |  |
| Baseline tumor size (cm) |  |  |  | 0.051 |  |  | 0.195 |
| ≤2 | 3 (2.2%) | 3 (2.2%) | 0 (0%) |  | 3 (2.2%) | 0 (0%) |  |
| >2, ≤4 | 23(16.7%) | 20 (14.5%) | 3 (2.2%) |  | 21 (15.2%) | 2 (1.4%) |  |
| >4 | 112(81.2%) | 72 (52.2%) | 40 (29%) |  | 86 (62.3%) | 26 (18.8%) |  |
| Tumor size after NACT (cm) |  |  |  | 0.091 |  |  | < 0.001 |
| ≤2 | 39(28.3%) | 31 (22.5%) | 8 (5.8%) |  | 37 (26.8%) | 2 (1.4%) |  |
| >2, ≤4 | 57(41.3%) | 40 (29%) | 17 (12.3%) |  | 49 (35.5%) | 8 (5.8%) |  |
| >4 | 42(30.4%) | 24 (17.4%) | 18 (13%) |  | 24 (17.4%) | 18 (13%) |  |
| Response to NACT |  |  |  | 0.019 |  |  | 0.013 |
| Responders | 78(56.5%) | 60 (43.5%) | 18 (13%) |  | 68 (49.3%) | 10 (7.2%) |  |
| Non-responders | 60(43.5%) | 35 (25.4%) | 25 (18.1%) |  | 42 (30.4%) | 18 (13%) |  |
| FIGO stage |  |  |  | < 0.001 |  |  | < 0.001 |
| IB | 30 (21.7%) | 30 (21.7%) | 0 (0%) |  | 30 (21.7%) | 0 (0%) |  |
| IIA | 40 (29.0%) | 40 (29%) | 0 (0%) |  | 40 (29%) | 0 (0%) |  |
| IIB | 6 (4.3%) | 6 (4.3%) | 0 (0%) |  | 6 (4.3%) | 0 (0%) |  |
| IIIC | 62(45.0%) | 19 (13.8%) | 43 (31.2%) |  | 34 (24.6%) | 28 (20.3%) |  |
| Tumor type |  |  |  | 0.155 |  |  | 0.924 |
| SCC | 120(87.0%) | 80 (58%) | 40 (29%) |  | 95 (68.8%) | 25 (18.1%) |  |
| Non-SCC | 18(13.0%) | 15 (10.9%) | 3 (2.2%) |  | 15 (10.9%) | 3 (2.2%) |  |
| Differentiation |  |  |  | 0.516 |  |  | 0.100 |
| Well and moderately | 92(66.7%) | 65 (47.1%) | 27 (19.6%) |  | 77 (55.8%) | 15 (10.9%) |  |
| Poorly | 46(33.3%) | 30 (21.7%) | 16 (11.6%) |  | 33 (23.9%) | 13 (9.4%) |  |
| Stromal invasion depth |  |  |  | 0.172 |  |  | < 0.001 |
| ≤1/3 | 33(23.9%) | 27 (19.6%) | 6 (4.3%) |  | 33 (23.9%) | 0 (0%) |  |
| >1/3, ≤2/3 | 59(42.8%) | 39 (28.3%) | 20 (14.5%) |  | 51 (37%) | 8 (5.8%) |  |
| >2/3 | 46(33.3%) | 29 (21%) | 17 (12.3%) |  | 26 (18.8%) | 20 (14.5%) |  |
| LVSI |  |  |  | 0.050 |  |  | < 0.001 |
| No | 96(69.6%) | 71 (51.4%) | 25 (18.1%) |  | 93 (67.4%) | 3 (2.2%) |  |
| Yes | 42(30.4%) | 24 (17.4%) | 18 (13.0%) |  | 17 (12.3%) | 25 (18.1%) |  |
| Perineural invasion |  |  |  | 0.570 |  |  | < 0.001 |
| No | 132(95.7%) | 92 (66.7%) | 40 (29%) |  | 109 (79%) | 23 (16.7%) |  |
| Yes | 6(4.3%) | 3 (2.2%) | 3 (2.2%) |  | 1 (0.7%) | 5 (3.6%) |  |
| Corpus involvement |  |  |  | 0.701 |  |  | 0.081 |
| No | 93(67.4%) | 65 (47.1%) | 28 (20.3%) |  | 78 (56.5%) | 15 (10.9%) |  |
| Yes | 45(32.6%) | 30 (21.7%) | 15 (10.9%) |  | 32 (23.2%) | 13 (9.4%) |  |
| Vaginal involvement |  |  |  | 0.467 |  |  | 0.834 |
| No | 127(92.0%) | 89 (64.5%) | 38 (27.5%) |  | 102 (73.9%) | 25 (18.1%) |  |
| Yes | 11(8.0%) | 6 (4.3%) | 5 (3.6%) |  | 8 (5.8%) | 3 (2.2%) |  |
| Incision margin |  |  |  | 1.000 |  |  | 0.053 |
| No | 136(98.6%) | 94 (68.1%) | 42 (30.4%) |  | 110 (79.7%) | 26 (18.8%) |  |
| Yes | 2(1.4%) | 1 (0.7%) | 1 (0.7%) |  | 0 (0%) | 2 (1.4%) |  |
| Parametrial involvement |  |  |  | 1.000 |  |  | 0.203 |
| No | 137(99.3%) | 94 (68.1%) | 43 (31.2%) |  | 110 (79.7%) | 27 (19.6%) |  |
| Yes | 1(0.7%) | 1 (0.7%) | 0 (0%) |  | 0 (0%) | 1 (0.7%) |  |
| Adjuvant radiation |  |  |  | 0.179 |  |  | < 0.001 |
| No | 79(57.2%) | 58 (42%) | 21 (15.2%) |  | 72 (52.2%) | 7 (5.1%) |  |
| Yes | 59(42.8%) | 37 (26.8%) | 22 (15.9%) |  | 38 (27.5%) | 21 (15.2%) |  |
| Adjuvant chemotherapy |  |  |  | NA |  |  | NA |
| No | 0(0%) | 0(0%) | 0(0%) |  | 0(0%) | 0(0%) |  |
| Yes | 138(100%) | 95(68.8%) | 43(31.2%) |  | 110(79.7%) | 28(20.3%) |  |

Abbreviations: LNr(-), negative radiological lymph node; LNr(+), positive radiological lymph node; LNp(-), negative pathological lymph node; LNp(+), positive pathological lymph node. NACT, neoadjuvant chemotherapy; FIGO, The International Federation of Gynecology and Obstetrics; SCC, squamous cell carcinoma; LVSI, lymph-vascular space invasion.
